# p53 Activation Effect in the Balance of T Regulatory and Effector Cell Subsets in Patients With Thyroid Cancer and Autoimmunity

**DOI:** 10.3389/fimmu.2021.728381

**Published:** 2021-08-30

**Authors:** Andrea Arena, Antonio Stigliano, Eugenia Belcastro, Ezio Giorda, Maria Manuela Rosado, Armando Grossi, Maria Rita Assenza, Fabiola Moretti, Alessandra Fierabracci

**Affiliations:** ^1^Infectivology and Clinical Trials Research Department, Children’s Hospital Bambino Gesù, Rome, Italy; ^2^Department of Clinical and Molecular Medicine, Azienda Ospedaliera-Universitaria S. Andrea, Sapienza University, Rome, Italy; ^3^Research Laboratories, Children’s Hospital Bambino Gesù, Rome, Italy; ^4^Department of Clinical Internal Sciences, Anesthesiology and Cardiovascular Sciences, Sapienza University of Rome, Rome, Italy; ^5^Unit of Endocrine Pathology of Post-Tumoral and Chronic Diseases, Children’s Hospital Bambino Gesù, Rome, Italy; ^6^Institute of Cell Biology and Neurobiology, National Research Council of Italy (CNR), Rome, Italy

**Keywords:** p53, T regulatory cells, T effector cells, thyroid cancer, thyroiditis, immunotherapy, pediatric disease

## Abstract

Carcinomas evade the host immune system by negatively modulating CD4+ and CD8+ T effector lymphocytes through forkhead box protein 3 (FOXP3) positive T regulatory cells’ increased activity. Furthermore, interaction of the programmed cell death 1 (PD1) molecule and its ligand programmed cell death ligand 1 (PDL1) inhibits the antitumor activity of PD1+ T lymphocytes. Immunotherapy has become a powerful strategy for tailored cancer patients’ treatment both in adult and pediatric patients aiming to generate potent antitumor responses. Nevertheless, immunotherapies can generate autoimmune responses. This study aimed to investigate the potential effect of the transformation-related protein 53 (p53) reactivation by a peptide-based inhibitor of the MDM2/MDM4 heterodimer (Pep3) on the immune response in a solid cancer, *i.e.*, thyroid carcinoma frequently presenting with thyroid autoimmunity. In peripheral blood mononuclear cell of thyroid cancer patients, Pep3 treatment alters percentages of CD8+ and CD4+ T regulatory and CD8+ and CD4+ T effector cells and favors an anticancer immune response. Of note that reduced frequencies of activated CD8+ and CD4+ T effector cells do not support autoimmunity progression. In evaluating PD1 expression under p53 activation, a significant decrease of activated CD4+PD1+ cells was detected in thyroid cancer patients, suggesting a defective regulation in the initial activation stage, therefore generating a protective condition toward autoimmune progression.

## Introduction

Tumor cells have the ability to evade the host immune system (1, 2). Immune escaping is a common feature of carcinomas and is achieved by multiple mechanisms, one of which is the negative modulation of CD4+ and CD8+ T effector (Teff) lymphocytes, caused by the activity of forkhead box protein 3 (FOXP3)-positive T regulatory cells (Treg) (3). Another mechanism occurs upon the interaction of the programmed cell death 1 (PD1) molecule and its ligand programmed cell death ligand 1 (PDL1), often expressed by tumor cells, which inhibits the antitumor activity of PD1+ Teff lymphocytes (4).

Remarkably, PDL1 overexpression has been detected in the microenvironment of different cancer conditions including mammary and colon adenocarcinomas (5), and PD1 is upregulated in tumor-infiltrating Treg and Teff cells, contributing to Teff exhaustion in tumor lymphocyte infiltration (1, 6). PD1 expression on CD8+ Teff and Treg indeed affects tumor regression, acting on their activity against tumor cells or immunosuppression, respectively (7). Furthermore, additional immune-suppressive receptors are upregulated in the cancer inflammatory microenvironment, *i.e.*, T cell immunoglobulin mucin 3 (TIM-3), cytotoxic T lymphocytes antigen 4 (CTLA-4), glucocorticoid-induced TNFR (tumor necrosis factor receptor)-related protein (GITR), and lymphocyte-activation gene 3 (LAG-3) (8). On a speculative basis, one can envisage that the overexpression of these molecules correlates with the higher suppressive activity of tumor-infiltrating Treg (8).

In light of the foregoing, immunotherapy has become a powerful clinical strategy for tailored treatment of cancer patients. Several approaches have been implemented to control the modulation of the immune system and generate potent antitumor immune responses with fewer side effects compared to standard chemotherapies and other drugs that directly kill cancer cells (9). Immunotherapies restore different physiological mechanisms, which are evaded during cancer progression, leading to the activation or boost of effector cells against cancer cells. Nevertheless, immunotherapies can face several challenges in terms of both efficacy and safety since these treatments can generate autoimmune responses in some patients with the destruction of healthy tissues (10). Regarding immunotherapy delivery systems, these include checkpoint inhibitors, *i.e.*, anti-PD1 (pembrolizumab, nivolumab, cemiplimab), anti-PDL1 (atezolizumab, avelumab, durvalumab), anti-PD1/PDL1 (AUNP12, under experimentation), and anti-CTLA-4 (ipilimumab) monoclonal antibodies, cytokines promoting lymphocyte activation, chimeric antigen receptor (CAR) and T cell receptor (TCR) engineered T cells, agonist antibodies against co-stimulatory receptor and cancer vaccines (9). In particular, PD1-PDL1 blocking antibodies can enhance the activity of tumor-specific Teff cells, modulate Treg function, and reduce anti-inflammatory interleukin (IL-10) while enhancing proinflammatory cytokines release (10). Interestingly, in the tumor microenvironment, the balance of PD1+ CD8+ Teff and Treg lymphocytes is a putative marker of the clinical response to PD1 blockade more reliable than expression of PDL1 or cancer mutation burden (7). Combined impairment of CTLA-4 and PD1 signal transduction showed efficacy especially in some patients affected by melanoma (11, 12). Sole inhibition of CTLA-4 has been recommended for the risk of toxicity and autoimmune manifestations, while PD1 blockage led to milder immunity against self [reviewed (rev) in (1)].

On exploring future avenues for cancer treatment especially aimed at ameliorating prognosis overall in the pediatric population, we envisage the potential use of p53 (transformation-related protein 53) reactivation (13, 14). p53 is a short-lived transcription factor part of a sequence-specific family including p63 and p73 (14). The activity of p53 in normal cells is under the control of two primary regulators, MDM2 (mouse double minute 2) and MDM4 (mouse double minute 4) proteins. MDM2 is a ubiquitin ligase able to bind p53. The subsequent p53 ubiquitination leads to its proteasome-dependent degradation. MDM2 activity is enhanced when it forms a heterodimer with MDM4 (15, 16). When p53 is detached from MDM4 and MDM2 or the heterodimer is dissociated, activation of the protein occurs (17). The protein, expressed at low concentration in various tissues, is found at high levels in damaged, tumoral, or inflamed conditions, playing a relevant regulatory function on gene expression [rev in (13, 17, 18)]. In human cancers, p53 mediates a crucial tumor suppression activity [rev in (13, 14)], by affecting different functional pathways: arrest cell cycle progression, program cell death including apoptosis and autophagy; induce senescence; alter metabolism, fertility, and stem cell development and regulation [rev in (13)]. p53 has also immune regulatory functions (19) by suppressing inflammation and autoimmunity in animal models and in humans (3, 19–21). In patients affected by rheumatoid arthritis (RA), which have lower p53 mRNA levels and higher percentages of circulating T helper 17 (Th17) cells compared to healthy controls, the inhibition of p53-MDM2 interaction by Nutlin-3a induced Treg differentiation under the effect of Th17 polarizing conditions (3). In Type 1 diabetes (T1D) patients, we proved that p53 activation increases the percentages of CD8+ Treg; however, CD8+ T cell subset was also increased, especially the frequency of activated Teff cells that may negatively influence disease progression (21). This further envisages that p53 activation immunotherapy cannot be administered to all categories of autoimmune disease patients or could be beneficial especially in conditions in which patients present a defect of wild-type p53 expression (3).

In this study, we aimed at investigating the consequences of p53 reactivation by Pep3, a peptide inhibitor of the MDM2/MDM4 heterodimerization (14), on the immune response in a model of solid cancer, *i.e.*, thyroid carcinoma that frequently occurs in association with thyroid autoimmunity (22, 23). The investigation was conducted to gain new insights and open immunotherapeutic perspectives to manage this condition both in adult and pediatric patients.

## Materials and Methods

### Subjects

Eleven patients affected by thyroid carcinoma (TC) were analyzed (**Table 1**). Within the group of TC patients recruited in the present study, the mean age was 51 years (ranging from 30 to 62 years, one male, 10 females). Subjects were enrolled in the follow-up for thyroid carcinoma with a mean of 9.7 years (yrs) of disease post-surgery (ranging from 6 months to 28 yrs) at the Department of Endocrinology, Azienda Ospedaliera-Universitaria S. Andrea, Sapienza University, Rome over the past 28 yrs. Sera from patients were analyzed for the presence of thyroglobulin (Tg) and thyroperoxidase (TPO) autoantibodies (AAbs) tested by chemiluminescence (ADVIA Centaur analyzer: Siemens Healthcare, Germany). All controls were matched to patients for sex, age, and ethnic and geographical origin. Recruited patients and controls were unrelated. The local Institutional Review Boards (IRB) of the Bambino Gesù Children’s Hospital (OPBG) and Sapienza University have approved the study, and all participants gave written informed consent in accordance with the Declaration of Helsinki.

**Table 1:** Demographic, clinical characteristics, and thyroid autoantibody levels in patients with thyroid carcinoma recruited for the study.

Pt	Sex	Actual age(years)	Thyroid carcinoma (TC) follow-upduration	TC histology	Associated diseases	Age of thyroiditis diagnosis	Familiarity	AAbs	*p53* codon 72genotype
1	Female	50.91	28 years	Papillary TC	AT	11	Hypertension	Tg pos (post- TX)	*Pro/*Pro
2	Female	60.26	17 years	Papillary TC Lymph node metastases (pT4b)	AT Endometrial cyst Ankylosing spondylitis	9	AT	Tg pos (pre- and post-TX)	*Pro/*Pro
3	Female	59.67	15 years	Papillary TC follicular variant (pT1N1pMx)	AT, bilateral hystero- annessiectomy Breast cancer	14	Hypothyroidism Breast cancer Cervical cancer Lung cancer	Tg pos (pre- TX)	*Arg/*Pro
4	Female	53.10	6 months	Papillary TC (pT1bpN1a)	AT Breast cancer Deep vein thrombosis	7	AT Melanoma Acute myocardial ischemia (AMI)	Tg pos (post- TX)	*Pro/*Pro
5	Female	60.99	1 year	Papillary TC (pT1bpNxpMx)	AT multiple myeloma	12		Tg pos TPO pos (post- TX)	*Pro/*Pro
6	Female	43.77	11 years	Papillary TC (pT3)Neck lymph node metastases	AT (histological diagnosis)	NA	Thyroid disease Hypertension	Tg neg TPO neg (pre- and post- TX)	*Pro/*Pro
7	Female	55.85	9 years	Papillary TC follicular variant (pT1)	AT Rheumatoid arthritis Vitiligo	13	Thyroid disease Colon carcinoma	Tg pos TPO pos (pre- TX) AAbs to citrullinated cyclic peptides	*Pro/*Pro
8	Female	29.42	3 years	Papillary TC (pT1)	AT Hereditary fructose intolerance Minor β thalassemia	11	AT Hypertension Prostate cancer	Tg pos TPO pos (pre- TX)	*Pro/*Pro
9	Male	56.93	1 year	Papillary TC follicular variant (pT1)	AT Melanoma	13	Thyroid carcinoma	Tg pos TPO pos (pre- TX)	*Pro/*Pro
10	Female	29.04	14 years	Papillary TC high cell variant (pT1)	AT	15	Thyroid carcinoma	Tg pos TPO pos (pre- TX)	*Pro/*Pro
11	Female	55.92	10 years	Papillary TC high cell variant (pT3pN1)	AT	13	Thyroid carcinoma	Tg pos TPO pos (pre- TX)	*Pro/*Pro

AAbs reference values: thyroperoxidase (TPO) <60 U/ml; thyroglobulin (Tg) 0–40 UI/ml; Pt, patient; AT, autoimmune thyroid disease; pos, positive; TX, thyroidectomy; NA, not available.

### Detection of p53 Codon 72 Polymorphism in Patients With Thyroid Cancer

Genomic leukocyte DNA was extracted from whole blood samples of patients by QIAmp DNA blood mini kit (Qiagen, Hilden Germany) according to the manufacturer’s guidelines. Polymerase chain reaction (PCR) was carried out with specific primers for exon 4 of the gene (Gene Bank ID:7157): forward 5’-AATGGATGATTGATGCTGTCCC-3’ and reverse 5’ GGTGCAAGTCACAGACTTGGC-3’ (24). The amplification lasted 35 cycles with 62°C annealing temperature. PCR sequencing was carried out with the BigDye Terminator v.3.1 Cycle sequencing protocol (Life Technologies, Applied Biosystems, Paisley, Scotland, UK). Products were then purified and sequenced with the Genetic Analyzer 3500 (Applied Biosystems HITACHI system).

### Cell Preparation and Culture Conditions

Peripheral blood mononuclear cells (PBMC) from TC patients and healthy donors (HD) were isolated from sodium heparinized venous blood samples (10 ml) using Ficoll-Hypaque (Histopaque, Sigma-Aldrich Chemical Company, St. Louis, MO, USA) and then frozen in liquid nitrogen according to standard protocols (25, 26). Liquid-nitrogen frozen TC and HD PBMC were quickly thawed in pre-warm RPMI medium (GibcoTM RPMI 1640 Medium, ThermoFisher Scientific, Waltham, MA, USA) supplemented with 10% fetal bovine serum (FBS, Hyclone, South Logan, UT, USA), L-glutamine (2 mM) (EuroClone S.p.A, Milan, Italy), penicillin (100 U/ml) (EuroClone S.p.A, Milan, Italy), and streptomycin (100 μg/ml) (EuroClone S.p.A, Milan, Italy) according to established protocols (26). Cells were centrifuged at 1,200 rpm for 5 min at room temperature (RT) and seeded into 48-well plates (Falcon, Corning 7 Incorporated, NY, USA) at density of 1.5×10^6^ cells per well in a final volume of 250 µl. Subsequently, the cells were treated with different doses of peptide 3 (Pep3) (10, 15, and 20 µM) or peptide 3 mutated (Pep3 MUT) (15 µM) as previously described (14). As reported in the literature, Pep3 is composed of 12 aminoacids and selectively impairs the MDM2/MDM4 heterodimer formation, thus activating the p53 function (14). Pep3 MUT has the same sequence of Pep3, except for a mutation in the key contact point of the peptide. The mutation, in turn, makes this peptide unable to impair the heterodimer formation; therefore, Pep3 MUT represents Pep3 specificity control for the experiment.

After 24 hours (h) of incubation, the cells were subsequently stimulated with the addition of Dynabeads Human T-activator CD3/CD28 (Life Technologies AS, Oslo, Norway) at a bead-to-cell ratio of 1:50, reflecting the physiological conditions and allowing the detection of immunomodulatory activity (25). The cells were harvested at 4 and 6 days after stimulation with anti-CD3/CD28 beads, and they were washed by centrifugation at 1,200 rpm for 5 min RT in phosphate-buffered saline (PBS, EuroClone S.p.A, Milan, Italy) before being used for flow cytometry analysis (FACS).

### Flow Cytometry Analysis

In order to analyze T-cell subsets, cells were stained as already described (27) for 20 min at 4°C for surface markers detection. The antibodies used are listed as follows: Brilliant Ultraviolet 737 (BUV737) conjugated mouse anti-human CD3 (Clone UCHT1; 1:40 dilution; BD Biosciences, CA, USA); Brilliant Violet 421 (BV421) conjugated mouse anti-human CD25 (Clone M-A251; 1:40 dilution; BD); allophycocyanin (APC) conjugated mouse anti-human CD8 (Clone RPA-T8; 1:10 dilution; BD); and R-phycoerythrin-Cyanine7 (PE-Cy7) conjugated mouse anti-human CD279 (programmed cell death 1, PD1) (Clone J105; 1:40 dilution; eBioscience, ThermoFisher Scientific). After incubation, cells were washed once with wash buffer (2% FBS in PBS) and centrifuged at 1,200 rpm for 5 min at RT. Subsequently, cells underwent FOXP3 intracellular staining using PE conjugated mouse anti-human FOXP3 antibody (Clone 259D/C7, BD) following the manufacturer’s protocol (Human FOXP3 Buffer Set, BD). According to literature, CD8+CD25+FOXP3+ cells were considered as CD8+ Treg and CD8+CD25−FOXP3− cells as CD8+ effector T cells (Teff) (28, 29). CD8+CD25+FOXP3− cells were considered activated CD8+ Teff cells. PD1+ cells within the total gate of CD8+ Treg and Teff were identified as CD8+ Treg PD1+ cells and CD8+Teff PD1+ cells. Moreover, in this study the same populations as described above were also evaluated among the CD4+ subset (**Figure S1**). Data were acquired using a Fortessa X-20 flow-cytometer [Becton and Dickinson (BD), Sunnyvale, CA, USA] and analyzed by FACSDiva software (BD Biosciences: San Jose, CA, USA). Dead cells were excluded from the analysis by side/forward scatter gating (**Figure S1**), and 50,000 events/sample were acquired on the lymphocyte gate.

### Statistical Analysis

Data are expressed as mean ± SEM. Statistical significance was evaluated among the treatments for all subsets analyzed in 11 TC patients by Wilcoxon matched-pairs signed rank test. Differences between HD and TC among the different treatments in terms of percentages of PD1+ cells among subsets analyzed were assessed by the Mann Whitney test. The analysis was carried out with the GraphPad Prism software version number 7.00 (GraphPad Software: San Diego, CA, USA). A result of p < 0.05 was considered statistically significant.

## Results

### Study Population

Demographic, clinical, and laboratory characteristics of patients are shown in **Table 1**. All subjects enrolled in the study are of Caucasian origin, coming from Central Italy. In addition to thyroid carcinoma, 10 patients (over 90% of total patients) also developed autoimmune thyroiditis (AT) and one patient (Pt) had AT histological diagnosis (Pt 6, **Table 1**). Among other autoimmune disorders, one patient was affected by ankylosing spondylitis (Pt 2, **Table 1**) and one patient by rheumatoid arthritis and vitiligo (Pt 7, **Table 1**). Associated cancer conditions included breast cancer (Pts 3–4, **Table 1**), multiple myeloma (Pt 5, **Table 1**), and melanoma (Pt 9, **Table 1**). Nine patients had family history for thyroid disorders; three patients (Pts 9–11, **Table 1**) had family history for thyroid cancer; one patient for breast cancer (Pt 3, **Table 1**), one patient for cervical cancer (Pt 3, **Table 1**), and one patient for colon cancer (Pt 7, **Table 1**). All patients are characterized by papillary thyroid cancer, a histotype associated with wt-p53 in almost 100% of cases (30). Since wtp53 with a polymorphism at codon 72 (*p53*P72) is associated with an increased risk of cancer due to the decreased ability of *p53*P72 to induce apoptosis (31), the polymorphism characterization was evaluated in the patients cohort of this study. Indeed, p53 *Arg/*Pro is present in one patient (Pt 3, **Table 1**), and the remaining 10 harbor the *Pro/*Pro genotype. Patients were recruited during the follow-up of thyroid carcinoma, which was diagnosed in adult age except for Pt 10, **Table 1**.

### Peptide 3 Efficacy on Thyroid Cancer PBMC Subsets

**Figure 1:**
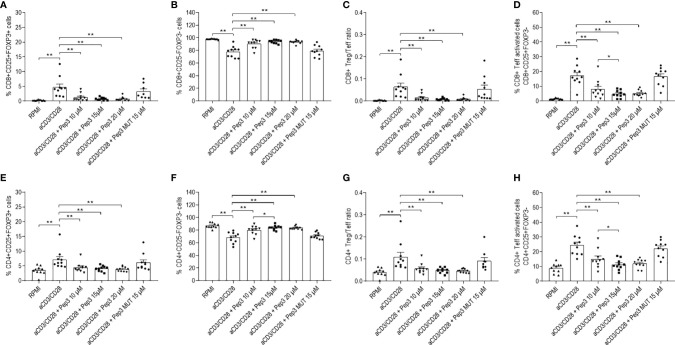
Frequency of T-cell populations in PBMC isolated from TC patients treated with peptide 3 and subsequently stimulated with anti-CD3/CD28 beads for 4 days. Flow-cytometry analysis of TC PBMC untreated unstimulated (RPMI) or stimulated (anti-CD3/CD28), treated with 10, 15, and 20 µM Pep3 or 15 µM of Pep3 MUT and subsequently stimulated with anti-CD3/CD28 beads for 4 days. Percentages of CD8+ subsets (CD8+ Treg, CD8+ Teff, CD8+ Teff activated) were calculated on total CD8+ cells. Percentages of CD4+ subsets (CD4+ Treg, CD4+ Teff, CD4+ Teff activated) were calculated on total CD4+ cells. Percentages of total CD8+ and CD4+ cells were calculated on total lymphocytes. Graphs show the percentages upon CD3/CD28 stimulation of CD8+ Treg as CD8+CD25+FOXP3+ cells **(A)**, CD8+ Teff as CD8+CD25−FOXP3− cells **(B)**, CD8+ Treg/Teff ratio **(C)**, CD8+ Teff activated cells as CD8+CD25+FOXP3− cells **(D)**, CD4+ Treg as CD4+CD25+FOXP3+ cells **(E)**, CD4+ Teff as CD4+CD25−FOXP3− cells **(F)** (please refer for gating strategy to **S1 Figure**), CD4+ Treg/Teff ratio **(G)**, percentage of CD4+ Teff activated cells as CD4+CD25+FOXP3− cells **(H)**. Statistical significance was evaluated using Wilcoxon matched-pairs signed rank test. Data are expressed as mean ± SEM, and each dot corresponds to an individual TC patient (n = 10). *p < 0.05, **p < 0.01.

In order to evaluate the effect of Pep3 in TC PBMC patients, different doses of Pep3, which been previously demonstrated efficacious (14), were used. In TC PBMC pretreated with Pep3 and subsequently stimulated for 4 days with anti-CD3/CD28 beads, the percentage of CD8+ Treg (CD8+CD25+FOXP3+) cells significantly decreased at the lowest dose used (10 μM) and did not further decrease at higher doses (15 and 20 μM) compared to untreated stimulated cells (**Figures 1A** and **S2**). As expected, the control peptide, Pep3 MUT, did not alter the frequency of CD8+ Treg cells, indicating the specific activity of Pep3. Conversely, the percentage of CD8+ Teff (CD8+CD25−FOXP3−) cells increased significantly upon Pep3 treatment (**Figures 1B** and **S2**); as a consequence, the ratio between the two subsets decreased (**Figure 1C**). Notably, the CD8+ Teff activated cell subset (CD8+CD25+FOXP3−) decreased significantly upon stimulation with anti-CD3/CD28 beads following pretreatment with the Pep3 at the same concentrations (**Figures 1D** and **S2**), meaning increased effect at 10 and 15 μM Pep3 treatment (**Figure 1D**). Again, no significant change was detected in cultures pretreated with Pep3 MUT at the dose of 15 μM, confirming the specificity of Pep3 activity (**Figures 1B–D**). Regarding CD4+ Treg (CD4+CD25+FOXP3+) cells, under the same conditions, percentages were decreased after Pep3 treatment (**Figure 1E**), while a significant increase was detected in CD4+ Teff (CD4+CD25−FOXP3−) cells (**Figure 1F**). Consequently, the CD4+ Treg/Teff ratio decreased after Pep3 treatment (**Figure 1G**). As observed above, CD4+ Teff activated cells (CD4+CD25+FOXP3−) significantly decreased in percentage and in a dose-dependent manner from 10 to 15 μM of Pep3 treatment (**Figure 1H**). These results indicate that in PBMC of patients affected by thyroid cancer, Pep3 treatment can alter the percentages of CD8+ and CD4+ Treg, CD8+ and CD4+ Teff cells, as well as of CD8+ and CD4+ Teff activated cells and favor an anticancer immune response. Importantly, the reduced frequencies of activated CD8+ and CD4+ Teff cells observed, *in vitro*, in PBMC of patients affected by thyroid cancer associated with autoimmune thyroiditis at time of analysis, do not foresee progression of autoimmune responses post-thyroidectomy *in vivo* during the follow-up.

**Figure 2:**
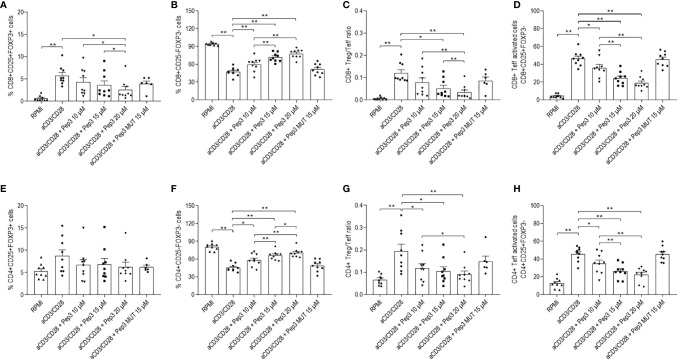
Frequency of T cell populations in PBMC from TC patients upon treatment with peptide 3 and subsequent stimulation with anti-CD3/CD28 beads for 6 days. Flow-cytometry analysis of TC PBMC following 10, 15, and 20 µM treatment relative to Pep3 and 15 µM treatment relative to Pep3 MUT and subsequent stimulation for 6 days with anti-CD3/CD28 beads. Graphs show the percentages upon CD3/CD28 stimulation of CD8+ Treg as CD8+CD25+FOXP3+ cells **(A)**, CD8+ Teff as CD8+CD25−FOXP3− cells **(B)**, CD8+ Treg/Teff ratio **(C)**, CD8+ Teff activated cells as CD8+CD25+FOXP3− cells **(D)**, CD4+ Treg as CD4+CD25+FOXP3+ cells **(E)**, CD4+ Teff as CD4+CD25−FOXP3− cells **(F)**, CD4+ Treg/Teff ratio **(G)**, CD4+ Teff activated cells as CD4+ CD25+FOXP3- cells **(H)**. Data are expressed as mean ± SEM, and each dot corresponds to an individual TC patient (n = 9). Statistical significance was evaluated using Wilcoxon matched-pairs signed rank test. *p < 0.05, **p < 0.01.

Effects of Pep3 exposure were still persistent after 6 days of stimulation with anti-CD3/CD28 beads, indicating the long-term activity of Pep3 on p53 activation (**Figures 2A–H**). Particularly, the effects on CD8+ Treg lowered but were still significant as at 4 days (**Figure 2A**). Effects on CD8+ Teff, CD4+ Teff (**Figures 2B, F**) and on activated CD8+ and CD4+ Teff (**Figures 2D, H**) cells were even higher compared to 4 days of treatment (**Figure S3**). Conversely, no effect was observed on CD4+ Treg (**Figure 2E**). As expected from these results, after 6 days, the relative ratios were substantially altered by Pep3 treatment (**Figure 2G**).

### Peptide 3 Efficacy on the Expression of PD1 Molecule in Thyroid Cancer PBMC Subsets

**Figure 3:**
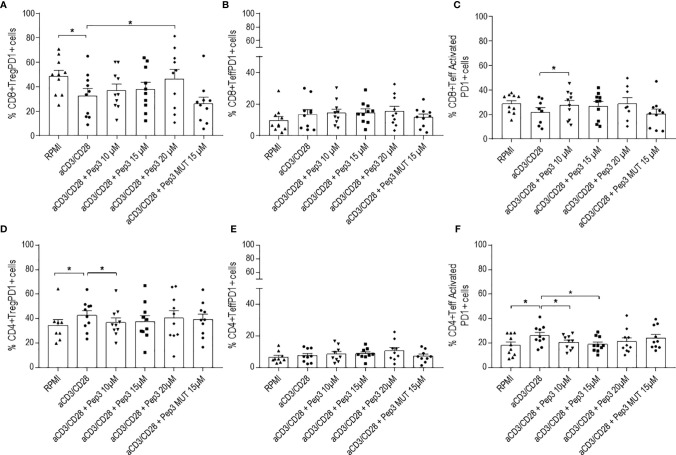
Frequency of CD8+PD1+ and CD4+PD1+ cell populations relative to thyroid cancer patients PBMC upon treatment with peptide 3 and subsequent stimulation with anti-CD3/CD28 beads for 4 days. Upper graphs show the percentages of CD8+ Treg PD1+ cells **(A)**, CD8+ Teff PD1+ cells **(B)**, CD8+ Teff activated PD1+ cells **(C)**. Lower graphs show the percentages of CD4+ Treg PD1+ cells **(D)**, CD4+ Teff PD1+ cells **(E)**, CD4+ Teff activated PD1+ cells **(F)**. Percentage of PD1+ cells was evaluated in comparison to the corresponding parental subset under evaluation. Statistical significance was evaluated using Wilcoxon matched-pairs signed rank test. Data are expressed as mean ± SEM, and each dot corresponds to an individual TC patient (n = 9-10). *p < 0.05.

**Figure 4:**
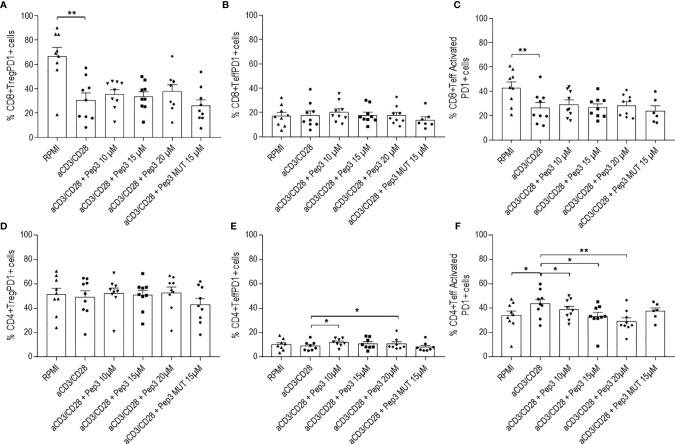
Frequency of CD8+PD1+ and CD4+PD1+ cell populations relative to thyroid cancer patients PBMC upon treatment with peptide 3 and subsequent stimulation with anti-CD3/CD28 beads for 6 days. Upper graphs show the percentages of CD8+ Treg PD1+ cells **(A)**, CD8+ Teff PD1+ cells **(B)**, CD8+ Teff activated PD1+ cells **(C)**. Lower graphs show the percentages of CD4+ Treg PD1+ cells **(D)**, CD4+ Teff PD1+ cells **(E)**, CD4+ Teff activated PD1+ cells **(F)**. Percentage of PD1+ cells was evaluated in comparison to the corresponding parental subset under evaluation. Statistical significance was evaluated using Wilcoxon matched-pairs signed rank test. Data are expressed as mean ± SEM, and each dot corresponds to an individual TC patient (n = 9). *p < 0.05 **p < 0.01.

With the purpose of investigating T cell activation following Pep3 reactivation, expression of the regulatory PD1 molecule was studied after 24 h of Pep3 treatment and subsequent anti-CD3/CD28 stimulation (**Figure 3A–F**, **4A–F**).

After 4 days of stimulation, Pep3 administration increased percentages of CD8+ Treg PD1+ cells (**Figure 3A**) and CD8+ Teff activated PD1+ (**Figure 3C**) at 20 and 10 µM, respectively, where no such effects were observed in CD8+ Teff PD1+ cells (**Figure 3B**).

Conversely, with regard to CD4+ T cells, Pep3 induced a decrease of CD4+ Teff activated PD1+ cells after 4 days of anti-CD3/CD28 stimulation (**Figure 3F**), while the total number of CD4+Teff PD1+ was not affected (**Figure 3E**). Pep3, at 10 µM, induced a reduction of the percentages of CD4+ Treg PD1+ (**Figure 3D**), although this effect did not persist until day 6 (**Figure 4D**).

After 6 days of stimulation, the effect of Pep3 on CD8+ Treg PD1+, CD8+ Teff PD1+, and CD8+ Teff activated PD1+ subsets was negligible (**Figures 4A–C**). Of note, a persistent reduction on the CD4+ Teff activated PD1+ in a more evident dose-dependent manner was detected (**Figure 4F**).

Consistently with the above results, comparing the effect of Pep3 pretreatment followed by 4 days of anti-CD3/CD28 stimulation in PBMC of TC cancer patients *versus* a small cohort of healthy donors (HD), no differences were observed in the analyzed cell subsets (**Figure S4**).

## Discussion

In the area of immunotherapy, immunological checkpoints, such as CTLA-4, PD1, or PDL1, are membrane proteins involved in the immune response that, when inhibited, cause an increase in T-cell activity and a consequent antitumor effect. However, these checkpoint inhibitors can also cause adverse effects including autoimmune endocrinopathies, such as thyroid dysfunction and hypophysitis (1). Therefore, in cancer patients receiving anti-PD1, anti-PDL1, or anti-CTLA4 drugs, regular endocrine assessment is recommended to make early diagnosis and appropriate treatment.

This study aimed to elucidate whether p53 reactivation by peptide 3 inhibitor of the MDM2/MDM4 heterodimer (14) formation, whose administration is known to inhibit tumor growth, could have beneficial effects on tumor progression by restoring antitumoral immune responses whilst avoiding the development of autoimmune responses.

The effect of p53 reactivator on immunotypes in the peripheral blood of patients affected by thyroid carcinoma was investigated. It is interesting to remark that treatment, indeed, caused a decrease of T regulatory CD4+ and CD8+ percentages and an increase of T effector cells either after 4 and 6 days of anti-CD3/CD28 stimulation following Pep3 pretreatment. Indeed, these data are strongly at odds with the anticipated outcome of activating p53, which should be an increase of FOXP3+ Tregs as observed in other experimental models of autoimmunity (3, 19, 20) and in PBMC of rheumatoid arthritis (3) and T1D patients (21). The current results of this investigation envisage the potential effect of the new immunotherapy in stimulating the activity of CD4+ and CD8+ antitumor specific T lymphocytes either in the tumor-infiltrating microenvironment and at distant sites in the periphery. As regard to the potential side effect associated with the use of other immune checkpoint targeted drugs such as PD1, PDL1, and CTLA4 toward autoimmunity, it is important to underline that in parallel to the reduction of the Treg/Teff ratio, percentages of activated T effector cells were decreased *in vitro*. This envisages that this potential immunotherapy drug has a lower risk of autoimmunity development and may potentially control an already established thyroid autoimmune disease such as Hashimoto’s thyroiditis (HT). HT is frequently observed in thyroid cancer patients, and it may precede the tumor development since pediatric age (32, 33), as observed in the pediatric patient 12 of the ongoing investigation (**Supplementary Table 1**). Of note, the results of the present study could indeed open new perspectives for the treatment of children affected by TC eventually associated with HT as observed in patients recruited at our Hospital (**Supplementary Table 1**), and thus they are presently object of examination.

To date the prognostic significance of PD1/PDL1 levels of expression in tumor cells, immune cells, and tumor microenvironment is controversial (34, 35). The PD1 immune checkpoint halts later immune responses primarily in periphery by attenuating T-cell signaling downstream of the T-cell receptor (36). This avoids immune cell damage in pathogenic responses and suppresses autoimmunity. Consistently, Nishimura (37) first highlighted a correlation between PD1 pathway and the onset of autoimmunity. In fact, a defective activation of regulatory T cells was observed in Type 1 diabetes patients due to the lower expression of PD1 (25). In multiple cancer cell types, PDL1 expression seems to be associated with the patient’s prognosis and be a predictive tool for the response to PD1/PDL1 therapy. Hence, high levels of PD1 and PDL1 correlate with a favorable prognosis while low PD1/PDL1 expression with poorer cancer survival (34). In other studies, PD-L1 and PD-1 expression was significantly correlated with several adverse prognostic pathologic factors, including higher T-stage, diffuse Lauren histologic type, and lymphatic invasion (33). Furthermore, recent studies highlight the importance of quantitatively imaging PD1/PDL1 interactions in tumor samples from patients. Analysis across multiple patient cohorts revealed inter-cancer, inter-patient, and intra-tumoral heterogeneity on interacting immune checkpoints (38). Sánchez-Magraner et al. reported that within tumors selecting for an elevated level of PD1/PDL1 interaction, there is a greater dependence on this pathway for immune evasion and they show more impressive patient response to treatment (38).

In light of the foregoing, the PD1 expression on lymphocyte subsets under the effect of p53 activation was additionally evaluated. Although, PD1+CD8+Treg cells increased after 4 days of anti-CD3/CD28 stimulation, no effect was observed at the following time of 6 days on CD8+ Treg, even if a significant increase of activated CD8+ Teff PD1+ at 4 days was observed although negligible at 6 days. Of note, PD1+CD4+Treg had a tendency to decrease after 4 days in favor of an antitumor immune response. Nevertheless, this effect did not persist in the following time point, 6 days post treatment. Interestingly, as regard the CD4+ counterpart, the decrease of activated CD4+ Teff included a decrease of PD1+ CD4+ activated Teff subset as compared to CD8+ Teff PD1+ subset, suggesting a defective regulation in the initial activation stage, therefore potentially protective toward the autoimmunity progression.

As regard the putative mechanism of the effect of Pep3, upon CD3/CD28 stimulation, to decrease CD4+Treg PD1+ cells, one may hypothesize a role of sterol-regulatory-element-binding protein (SREBP) cleavage-activating-protein (SCAP) signaling that has been shown to link TCR engagement to PD1 expression (39). Moreover, SREBP/SCAP pathway controls the expression of enzymes for both fatty-acid synthesis and mevalonate metabolism. Thus, drugs such Simvastatin, which target the enzyme 3-hydroxy-3-methylglutaryl-CoA reductase (HMGCR) and the geranylgeranyl transferase type I inhibitor (GGT1)-2147 of PD1, could modulate PD1 expression on CD4+Treg. Further, p53 is known to repress the mevalonate pathway (40).

On a general ground, the molecular mechanisms underlying Pep3 activity on different target cells and in particular on T cells remain to be fully elucidated. The transcriptional program elicited by p53 differs depending on the cell type, activation status of the cells, and the microenvironment. In immune cells, FOXP3 represents a well-defined target of p53 (41). In a preliminary evaluation of the transcriptional program following Pep3 treatment on human healthy PBMC, a set of genes that are directly and indirectly related to p53 activity and involved in tumor and immune cell activation was investigated. Initial data showed increased mRNA levels of *FOXP3* and *IL 1β*, a NF-kB target and a marker of antitumoral response (42), while no remarkable change of NF*-kB1 mRNA* levels, a Rel protein-specific transcription inhibitor, was observed. Although these results were achieved with a small number of samples and additional experiments are required to provide a clear and strong evidence, they may indicate a specific activity of p53 re-activation by Pep3 in HD PBMCs.

In conclusion, the present findings indicate the potential utility of p53 reactivation from a therapeutic perspective for treatment of autoimmunity and associated conditions of cancer and autoimmune diseases.

## Data Availability Statement

The raw data supporting the conclusions of this article will be made available by the authors, without undue reservation.

## Ethics Statement

The studies involving human participants were reviewed and approved by the Local Institutional Review Board (IRB) of the Bambino Gesù Children’s Hospital (OPBG) and Sapienza University. The patients/participants provided their written informed consent to participate in this study.

## Author Contributions

AA, EG, and MRA conducted experiments. AS provided materials and clinical informations on patients. FM designed the molecular analysis and contributed to the design of the study and drafting the whole manuscript. EB and MMR critically revised the study and contributed to drafting of the manuscript. AG added clinical informations on pediatric patients. AF designed and supervised conduction of the study and wrote the main manuscript. All authors reviewed the manuscript. All authors contributed to the article and approved the submitted version.

## Funding

This work was supported by the Italian Ministry of Health Ricerca Corrente RC2020_INFETT_FIERABRACCI (AF) and by the Italian Association for Cancer Research Grant AIRC IG 21814 (FM).

## Conflict of Interest

The authors declare that the research was conducted in the absence of any commercial or financial relationships that could be construed as a potential conflict of interest.

## Publisher’s Note

All claims expressed in this article are solely those of the authors and do not necessarily represent those of their affiliated organizations, or those of the publisher, the editors and the reviewers. Any product that may be evaluated in this article, or claim that may be made by its manufacturer, is not guaranteed or endorsed by the publisher.
