# Supplementary material for: p53 Activation Effect in the Balance of T Regulatory and Effector Cell Subsets in Patients With Thyroid Cancer and Autoimmunity
Source: Front Immunol. 2021 Aug 30;12:728381. doi: 10.3389/fimmu.2021.728381 (PMC8442659; doi:10.3389/fimmu.2021.728381)
Supplement: Supplementary file 1 [file DataSheet_1.pdf]

Supplementary Figures and Table

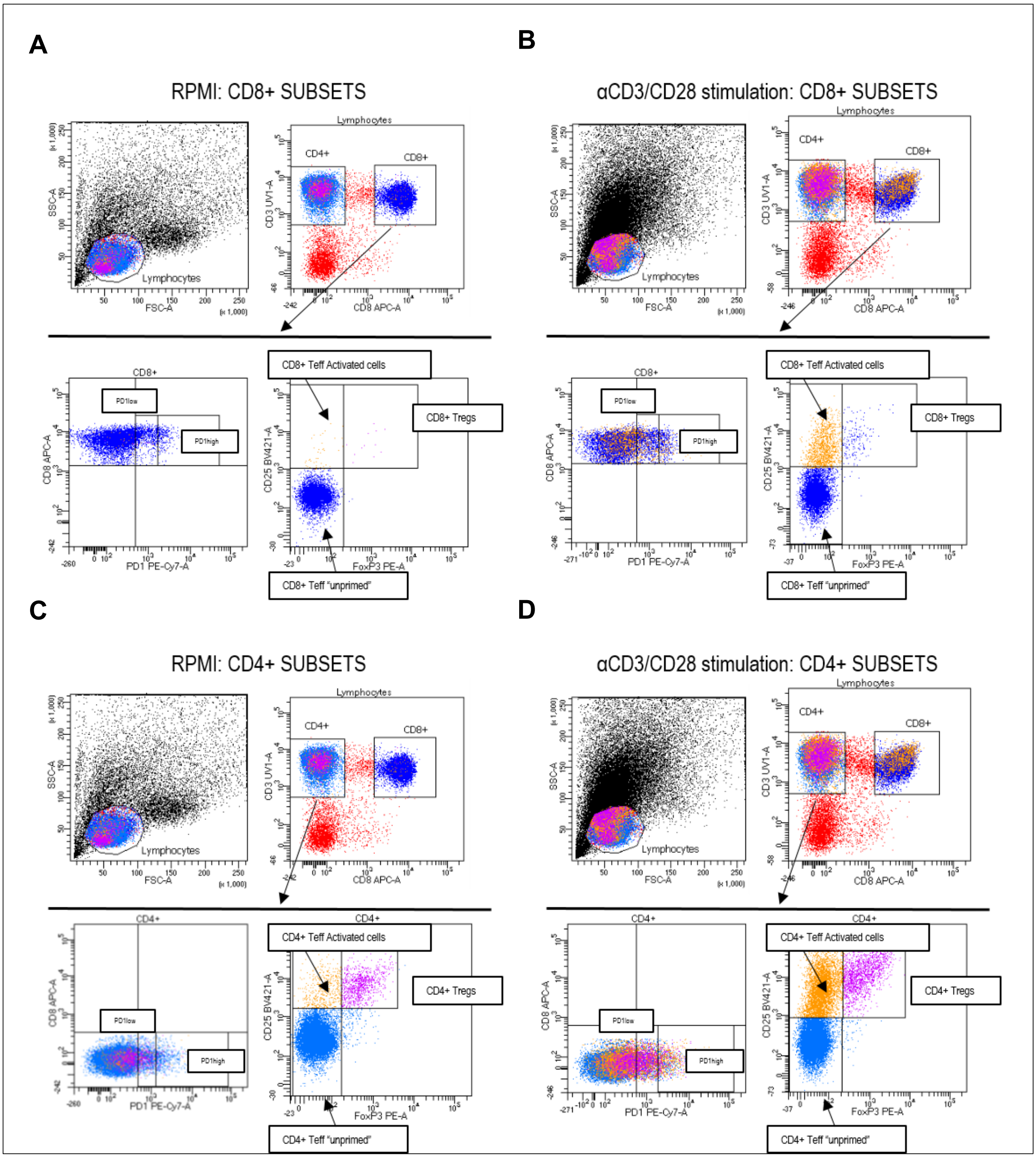

S1 Fig. Gating strategy used in the flow-cytometry analysis to define CD8+ and CD4+ T cell subsets. Lymphocytes were gated through their forward/scatter properties (FSC-A/SSC-A plot). 50,000 lymphocytes were acquired. In this example, nitrogen frozen TC PBMC were thawed, stained as described in the method section for antibodies to CD3, CD8, CD25, PD1 and FOXP3, and subsequently analyzed. The plots show CD8+ subsets, CD8+ Treg, CD8+ Teff, CD8+ Teff activated and the analysis of PD1+, in RPMI (**A**) and after anti-CD3/CD28 stimulation (**B**); plots

show also the CD4+subsets, CD4+ Treg, CD4+ Teff, CD4+ Teff activated and the analysis of PD1+, in RPMI (**C**) and after anti-CD3/CD28 stimulation (**D**).

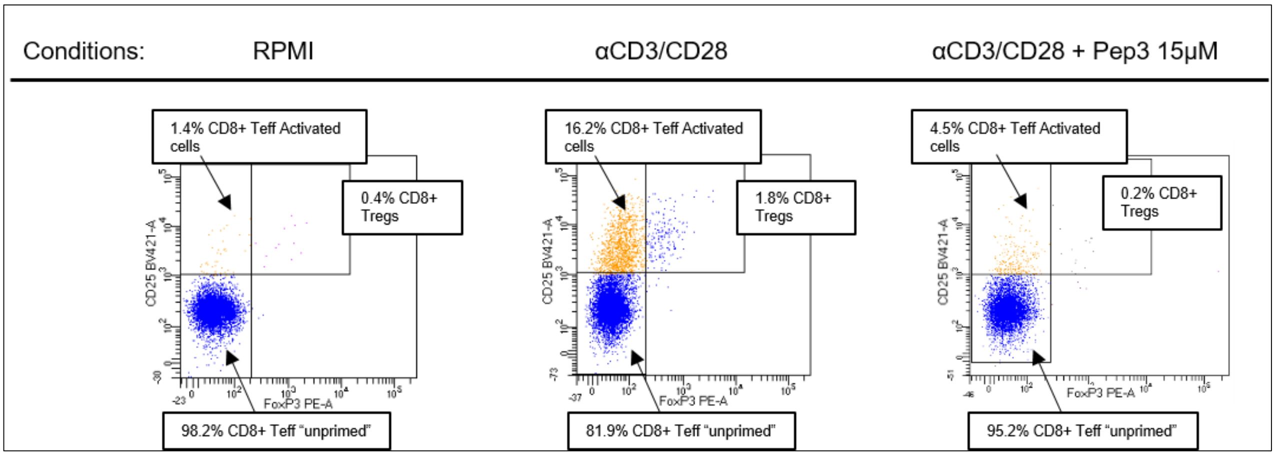

S2 Fig. Representative patient CD8+ subsets after Pep3 treatment. Representative plots indicate and compare the percentages of CD8+ Treg, CD8+ Teff and CD8+ activated Teff among RPMI, anti-CD3/CD28 stimulated and anti-CD3/CD28 stimulated cells pre-treated with 15 μM of Pep3.

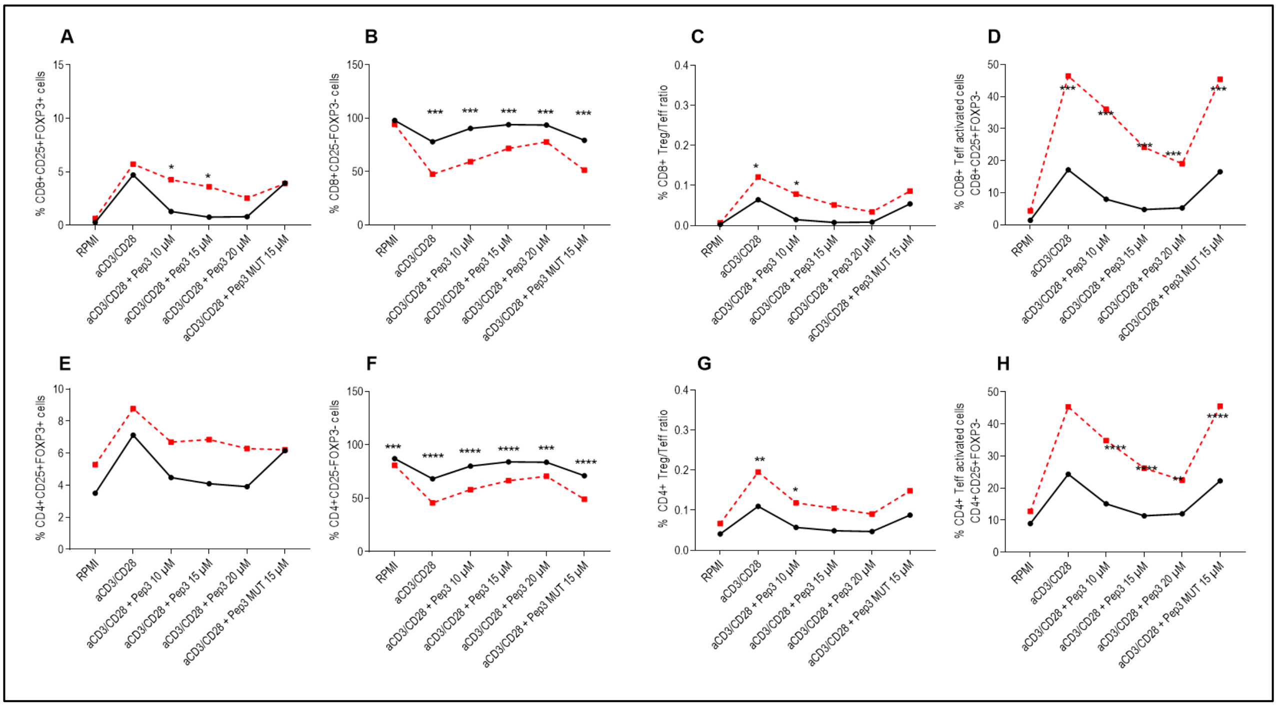

S3 Fig. Comparison of the frequency of T cell populations in PBMC isolated from patients affected by thyroid cancer after treatment with peptide 3 and subsequent stimulation with anti-CD3/CD28 beads for 4 days or for 6 days. Graphs show the percentages upon CD3/CD28 stimulation of the frequency of CD8+ Treg as CD8+ CD25+FOXP3+ cells (**A**), CD8+ Teff as CD8+ CD25-FOXP3- cells (**B**), CD8+ Treg/Teff ratio (**C**), frequency of CD8+ Teff activated cells as CD8+ CD25+FOXP3- cells (**D**), CD4+ Treg as CD4+ CD25+FOXP3+ cells (**E**), CD4+ Teff as CD4+ CD25-FOXP3- cells (**F**), CD4+ Treg/Teff ratio (**G**), frequency of CD4+ Teff activated cells as CD4+ CD25+FOXP3- cells (**H**). Difference among the various cell populations analyzed were

tested for statistical significance using unpaired t test (Mann Whitney test). Data are expressed as mean ± SEM of TC patients (n = 10, 4 days, black line) and TC patients (n = 9, 6 days, dotted line) * p<0.05, ** p<0.01, *** p<0.001, **** p<0.0001.

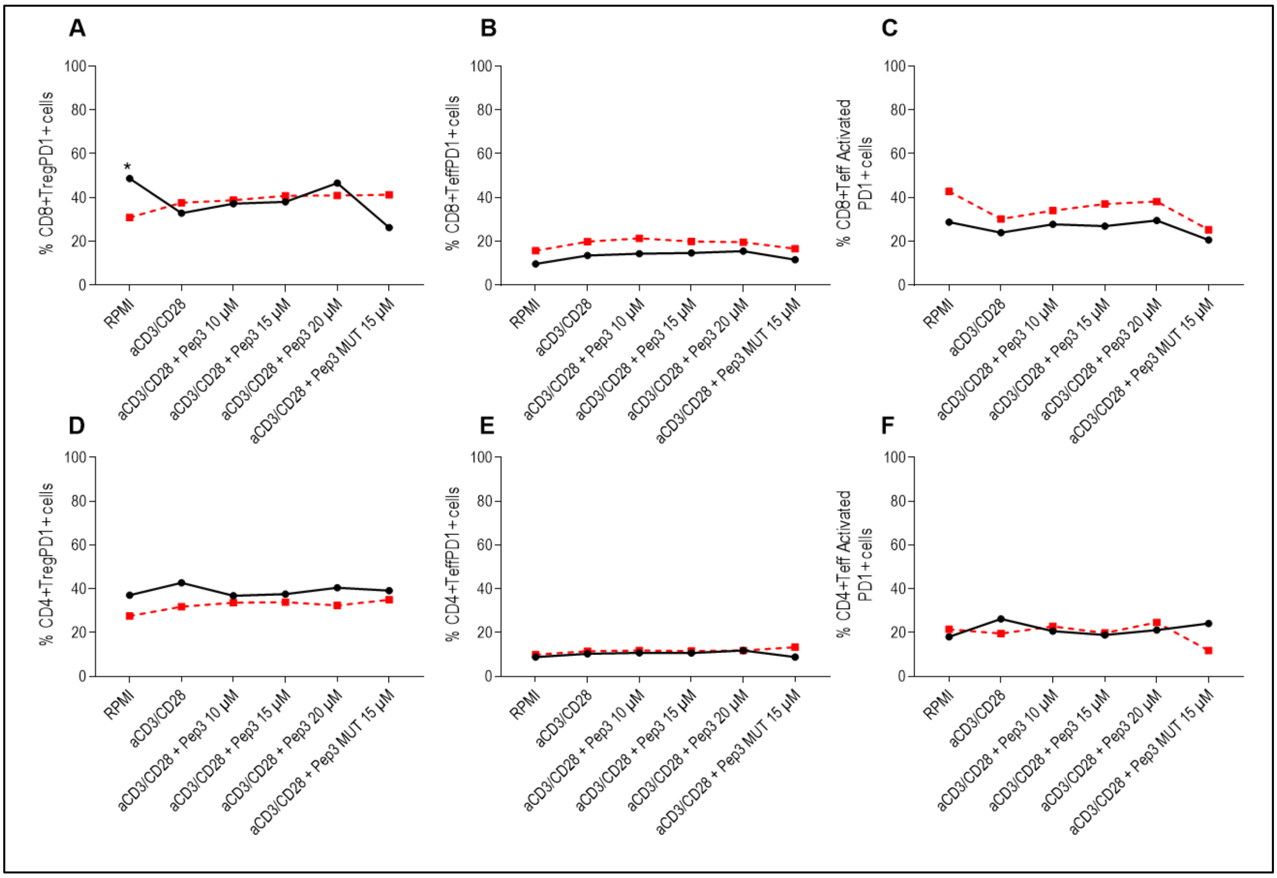

S4 Fig. Frequency of CD8+PD1+, CD4+PD1+, CD8+ and CD4+ Teff activated PD1+ cell populations in PBMC isolated from thyroid cancer patients and healthy donors (HD) after treatment with peptide 3 and subsequent stimulation with anti-CD3/CD28 beads for 4 days. Upper graphs show the percentages upon CD3/CD28 stimulation of CD8+ Treg PD1+ cells (**A**), CD8+ Teff PD1+ cells (**B**) and CD8+ Teff activated PD1+ cells (**C**). Lower graphs show the percentages upon CD3/CD28 stimulation of CD4+ Treg PD1+ cells (**D**), CD4+ Teff PD1+ cells (**E**) and CD4+ Teff activated PD1+ cells (**F**). PD1+ cells were evaluated in comparison to the corresponding parental subset under evaluation. Statistical significance was evaluated using unpaired t test (Mann Whitney test). Data are expressed as mean ± SEM of HD (n = 14, dotted line) and TC patients (n= 9-10, black line).* p<0.05.

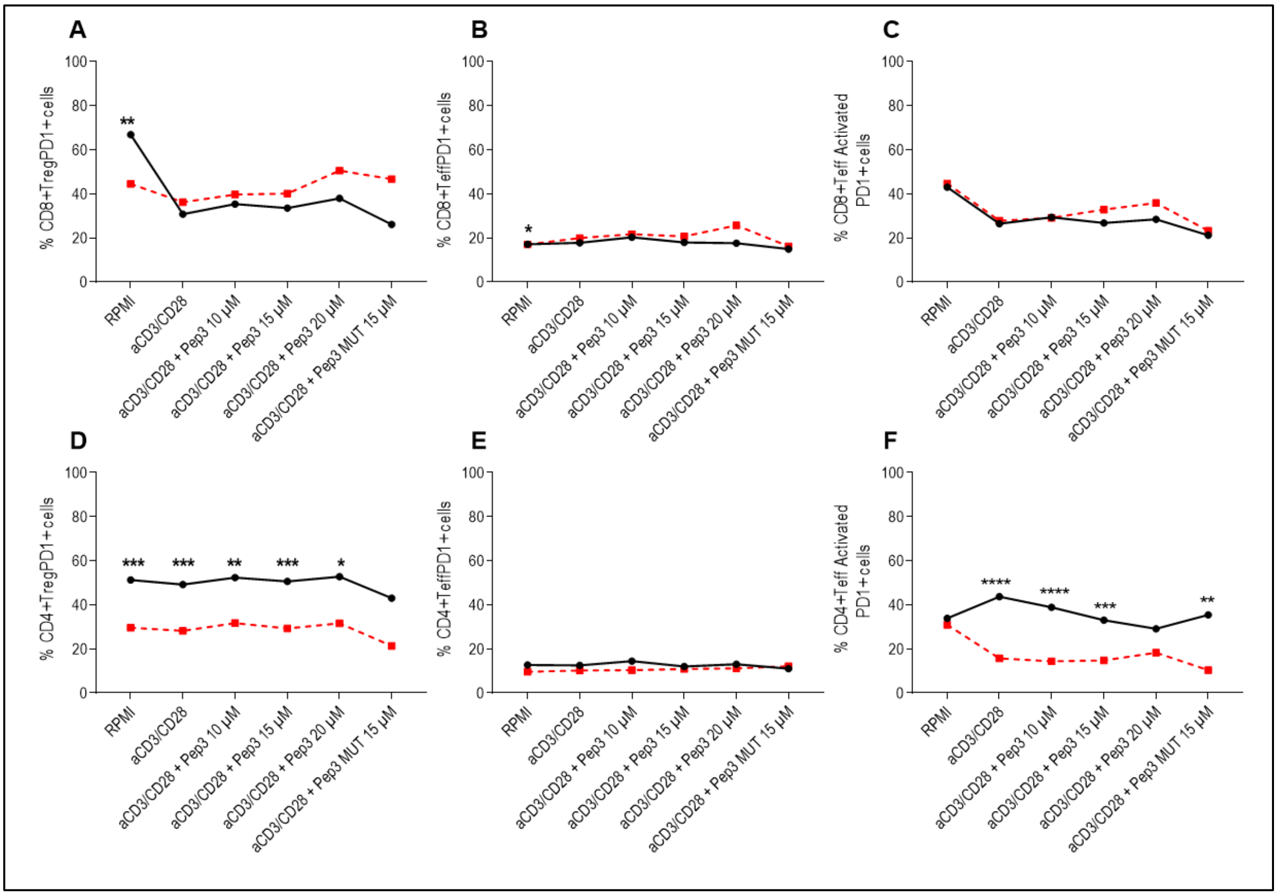

S5 Fig. Frequency of CD8+PD1+, CD4+PD1+, CD8+ and CD4+ Teff activated PD1+ cell populations in PBMC isolated from thyroid cancer patients and healthy donors (HD) after treatment with peptide 3 and subsequent stimulation with anti-CD3/CD28 beads for 6 days. Upper graphs show the percentages upon CD3/CD28 stimulation of CD8+ Treg PD1+ cells (**A**), CD8+ Teff PD1+ cells (**B**) and CD8+ Teff activated PD1+ cells (**C**). Lower graphs show the percentages upon CD3/CD28 stimulation of CD4+ Treg PD1+ cells (**D**), CD4+ Teff PD1+ cells (**E**) and CD4+ Teff activated PD1+ cells (**F**). PD1+ cells were evaluated in comparison to the corresponding parental subset under evaluation. Statistical significance was evaluated using unpaired t test (Mann Whitney test). Data are expressed as mean ± SEM of HD (n = 14, dotted line) and TC patients (n= 9-10, black line).* $p<0.05$, ** $p<0.01$, *** $p<0.001$, **** $p<0.0001$.

S1 Table. Demographic, clinical characteristics and thyroid autoantibodies levels in a group of pediatric patients with thyroid carcinoma referred at Children's Hospital Bambino Gesù.

Pt	Sex	Actual Age (years)	Follow-up duration	Thyroid carcinoma (TC) histology	Associated Diseases	Familiarity	Thyroid AAbs
1	F	17	4 months	Multifocal papillary TC diffuse sclerosant variety (pT1bmN1b)	AT		Tg neg (post-TX)
2	F	20.1	1.4 years	Papillary TC on ectopic thyroid tissue	Thyroid duct cyst		Tg neg (post-TX)
3	M	20.4	4 months	Multifocal papillary TC tall cell type (15%)	Previous HL previous BMT Hypergonadotropic hypogonadism	Heart disease	Tg neg (post-TX)
4	F	17.6	4 months	Papillary TC solid variant (60%) (pT4am, pN1a)			Tg neg (post-TX)
5	F	13.9	4 months	Papillary TC (pT2mNxMx – Stage I)			Tg neg (post-TX)
6	M	14.9	4 years	Multifocal Papillary TC (pT2 pN1b pMx. Stage 1)			Tg neg (post-TX)
7	M	7.6	9 years	Papillary TC (pT4a pN1b pMx Stage I)			Tg neg (post-TX)
8	M	11.5	6.25 years	Multifocal papillary TC (pT3mN1aMx Stage 1)	AT		Tg pos (700 IU/ml) (post-TX)
9	F	10.1	5.3 years	Papillary TC (pT3m N1b Mx)			Tg neg (post-TX)
10	F	17	4.8 years	Multifocal papillary TC			Tg neg (post-TX)
11	M	9	4 years	Multifocal papillary TC (pT3m)			Tg neg (post-TX)
12	F	16.3	6.4 years	Multifocal papillary TC (pT1am)	Turner syndrome AT Hypogonadism Left ventricular hypoplasia		Tg pos (51.3 IU/ml) (post TX)
13	M	12.9	9.8 years	Multifocal papillary TC (pT3mpN1b pMx Stage I)	AT	AT Graves' disease	Tg pos (> 500 IU/ml) TPO pos (223.5 U/mL) (post TX)

AAbs reference values: thyroperoxidase (TPO) < 60 U/mL; thyroglobulin (Tg) 0-40 UI/mL. Pt: patient; AT, autoimmune thyroid disease; Pos: positive; TX: thyroidectomy; Neg: negative; BMT, bone marrow transplantation; HL Hodgkin lymphoma.
